# Acute-on-chronic placental abruption complicated by prolonged oligohydramnios and life-threatening hemorrhagic shock: a case report

**DOI:** 10.1097/RC9.0000000000001038

**Published:** 2026-09-23

**Authors:** Zain Eddin Alhawari, Dema Adwan

**Affiliations:** a Faculty of Medicine, Damascus University, Damascus, Syria; b Department of Obstetrics and Gynecology, University Hospital of Obstetrics and Gynecology, Damascus University, Damascus, Syria

**Keywords:** acute-on-chronic placental abruption, Couvelaire uterus, emergency cesarean, placental insufficiency, uterine atony, case report

## Abstract

**Introduction and importance:**

Acute-on-chronic placental abruption is a volatile, high-risk obstetric emergency that poses severe diagnostic and management challenges. In its chronic phase, persistent decidual hemorrhage can progressively impair placental function, subsequently leading to amniotic fluid depletion and prolonged oligohydramnios. This condition can remain misleadingly compensated for months, masking severe underlying placental insufficiency before triggering catastrophic maternal-fetal decompensation.

**Case presentation:**

A 34-year-old pregnant woman with no chronic illnesses presented with an exceptional 2-month history of recurrent, idiopathic vaginal bleeding starting at 12 weeks of gestation, which necessitated a total of 6 units of fully cross-matched packed red blood cells. Emergency ultrasonography upon arrival demonstrated severe oligohydramnios and an organizing retroplacental hematoma, while the fetus maintained stable viability and normal cranial growth (BPD 65.8 mm). At 28 weeks, she experienced a sudden, catastrophic acute-on-chronic abruption event, presenting in profound, life-threatening hemorrhagic shock with a critical hemoglobin drop to 7.1 g/dL. Given her life-threatening hemodynamic instability, an immediate emergency exploratory laparotomy and cesarean section were performed as a lifesaving measure. Intraoperatively, significant hemorrhage due to severe uterine atony occurred, mimicking a Couvelaire uterus, and was successfully managed using a uterine compression suture and aggressive cross-matched transfusions. Post-delivery gross pathological evaluation of the placenta supported the clinical chronology, revealing a notable dual-generation clot pathology consisting of old organized dark clots interspersed with fresh bright red clots. The patient was monitored closely for 10 days postoperatively and was discharged in stable health; she is currently undergoing regular follow-up in the outpatient clinic.

**Discussion:**

This case highlights the extreme maternal-fetal morbidity associated with an acute abruption superimposed on a chronic detachment. Aggressive hematological support and tranexamic acid may stabilize maternal parameters temporarily but can obscure the progressive, unstable nature of the retroplacental space, creating a false sense of security. When severe oligohydramnios persists and transfusion requirements escalate, a lower threshold for early termination of pregnancy should be favored to safeguard maternal life over high-risk, prolonged conservative measures. Macroscopic placental pathology provides a vital diagnostic map to substantiate acute-on-chronic abruption events and justify intraoperative surgical decisions, such as selecting an open laparotomy over minimally invasive approaches in emergency situations.

**Conclusion:**

Acute-on-chronic placental abruption carries an unpredictable risk of sudden, severe maternal-fetal morbidity. Clinicians must remain extremely vigilant, as stable chronic placental separation can acutely rupture into a life-threatening obstetric shock. Macroscopic evaluation of dual-generation placental clots serves as a valuable pathological record to confirm the clinical chronology and guides immediate, aggressive surgical preparedness.

## Introduction

Placental abruption is typically defined as the premature detachment of a normally implanted placenta from the uterine wall before the delivery of the fetus^[^1^]^. This is usually associated with adverse perinatal outcomes, especially when it starts in the early stages of pregnancy, which leads to a more aggressive disease course^[^2^]^. Managing this condition is a major obstetric challenge, particularly when it is latent or chronic. It is a risk factor for Rhesus-negative patients with Rhesus alloimmunization^[^3^]^.

HIGHLIGHTS

- Acute-on-chronic placental abruption is a volatile, high-risk obstetric emergency.
- Persistent intrauterine bleeding can progressively lead to severe oligohydramnios.
- A catastrophic acute exacerbation can abruptly precipitate profound hemorrhagic shock.
- Emergent laparotomy and cross-matched transfusion are crucial for maternal survival.
- Aggressive conservative management may mask an unstable retroplacental hematoma.

We report an exceptional case of acute-on-chronic placental abruption that presented with prolonged oligohydramnios lasting 2 months, complicated by a severe maternal hemoglobin (Hb) drop to 7.1 g/dL and by profound hemorrhagic shock. This case highlights the extreme morbidity associated with an acute exacerbation superimposed on a chronic abruption and underscores the critical need for timely diagnosis, rapid surgical intervention, and aggressive resuscitation.

The work has been reported in line with the SCARE 2025 guidelines criteria.

## Case presentation

A 34-year-old nonsmoking, non-alcoholic woman with no chronic medical conditions was admitted with a singleton pregnancy. Her obstetric history included three previous vaginal deliveries: two healthy live births and one neonatal death due to hydrocephalus and spina bifida. The patient’s condition before pregnancy was stable, and her Hb level was 9.1 g/dL (Table 1).

**Table 1:** Laboratory findings at key time points.

	Before pregnancy (baseline)	At bleeding episode (6 units transfusion)	At emergency cesarean section	At discharge (postoperative day 10)
Hb (12–17 g/dL)	9.1	6.7	7.1	9.9
RBC count (4–5 × 10^6^/µL)	3.9	2.48	2.58	3.43
HCT (36–52%)	29.0	20.2	21.7	29.1
PLT (150–450 × 10^3^/µL)	206	272	270	199

Hb, hemoglobin; RBC, red blood cell; HCT, hematocrit; PLT, platelets.

In the current pregnancy, idiopathic vaginal bleeding began at 12 weeks of gestation. She received tocolytics, vitamin K, and tranexamic acid. Despite this, the intensity of bleeding progressively increased. This persistent blood loss necessitated a total of 6 units of fully cross-matched packed red blood cells, which were carefully matched to the patient’s blood group and Rh factor and were administered over three separate transfusion episodes (Table 1). The patient had irregular antepartum follow-up at external regional facilities, without detailed serial ultrasonographic records available. Upon emergency presentation to our center at 28 weeks of gestation with severe vaginal bleeding and hemorrhagic shock, immediate bedside ultrasonography revealed marked oligohydramnios, a retroplacental hematoma, and a singleton fetus with good fetal movements and a normal heart rate. The chronic nature of the pathology was clearly documented via ultrasound, which revealed an organizing retroplacental and subchorionic hematoma alongside a normal biparietal diameter (BPD) of 65.8 mm and distinct fetal crowding (Fig. 1a–d).

**Figure 1.**
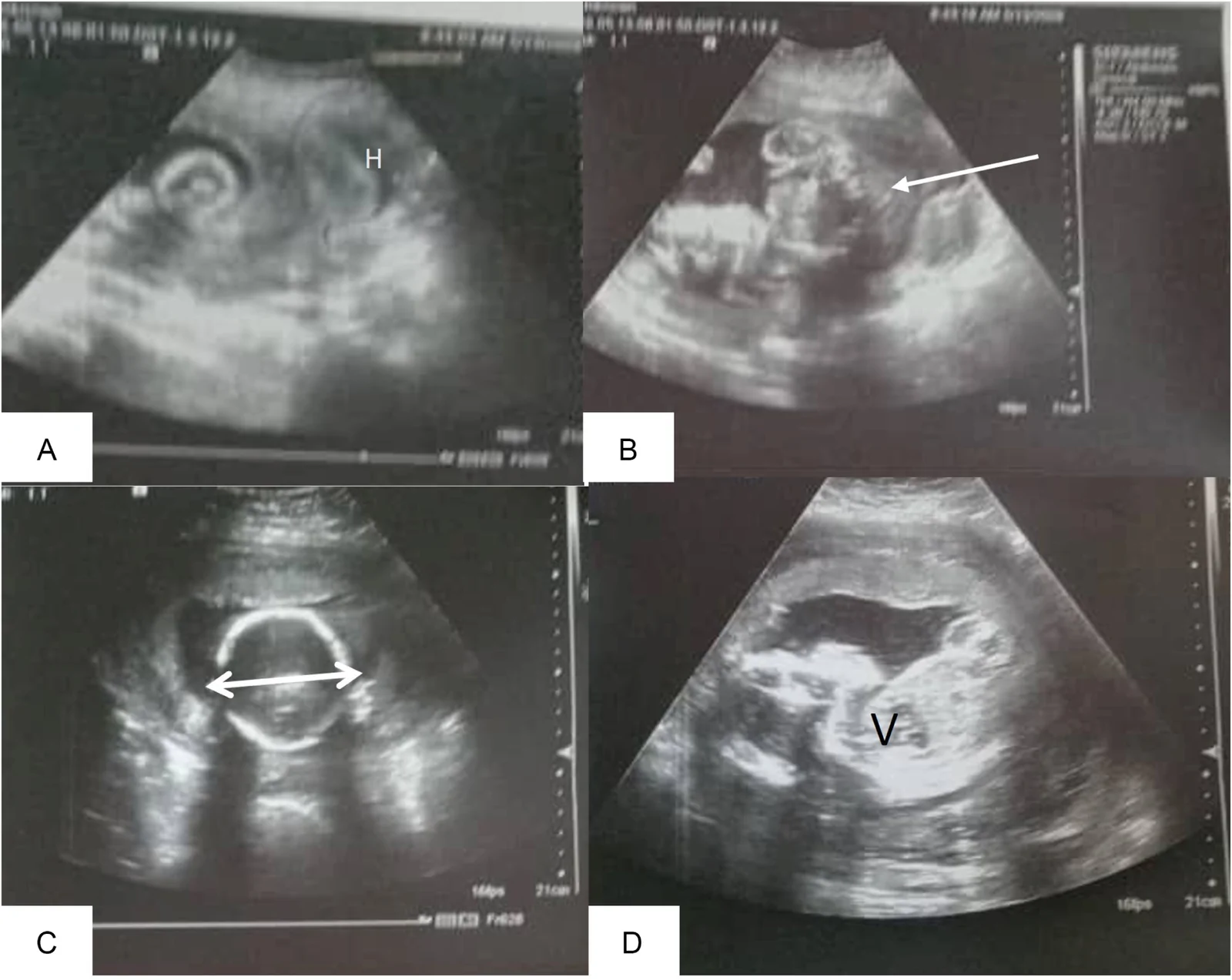
Ultrasonographic features of chronic placental abruption and associated oligohydramnios. (A) Retroplacental and subchorionic collection with heterogeneous echogenicity. The label (H) indicates an organizing, partially liquefied chronic retroplacental hematoma of a 2-month duration, which induced persistent placental separation. (B) Oblique scan demonstrates severe amniotic fluid depletion. The white arrow highlights the absence of amniotic fluid pockets and marked fetal crowding due to the large chronic hematoma encroaching upon the gestational sac. (C) Transverse view of the fetal head shows a normal biparietal diameter (BPD) of 65.8 mm. The double-headed arrow confirms appropriate gestational growth and head circumference symmetry despite severe long-standing placental insufficiency. (D) Sagittal scan confirms persistent fetal viability. The label (V) indicates the fetal chest area where active somatic movements and regular cardiac activity were documented during the evaluation.

At 28 weeks of gestation, the patient developed severe uterine contractions accompanied by worsening vaginal bleeding and the passage of clots. She rapidly progressed to hemorrhagic shock with hypotension, tachycardia, and a hemoglobin level of 7.1 g/dL (Table 1). Given the patient’s life-threatening hemodynamic instability, an immediate exploratory laparotomy and emergency cesarean section were performed as a lifesaving measure to provide rapid surgical access and secure hemostasis (Fig. 2a). During the procedure, macroscopic examination of the delivered placenta confirmed the clinical course, displaying distinct areas of old, dark, organized clots mixed with fresh, bright red clots (Fig. 3). A live female neonate was delivered at 28 weeks’ gestation, weighing 900 g. She presented with respiratory distress syndrome (RDS) and severe hypoxemia, resulting in irreversible brain injury; she was placed in an incubator for neonatal intensive care.

**Figure 2.**
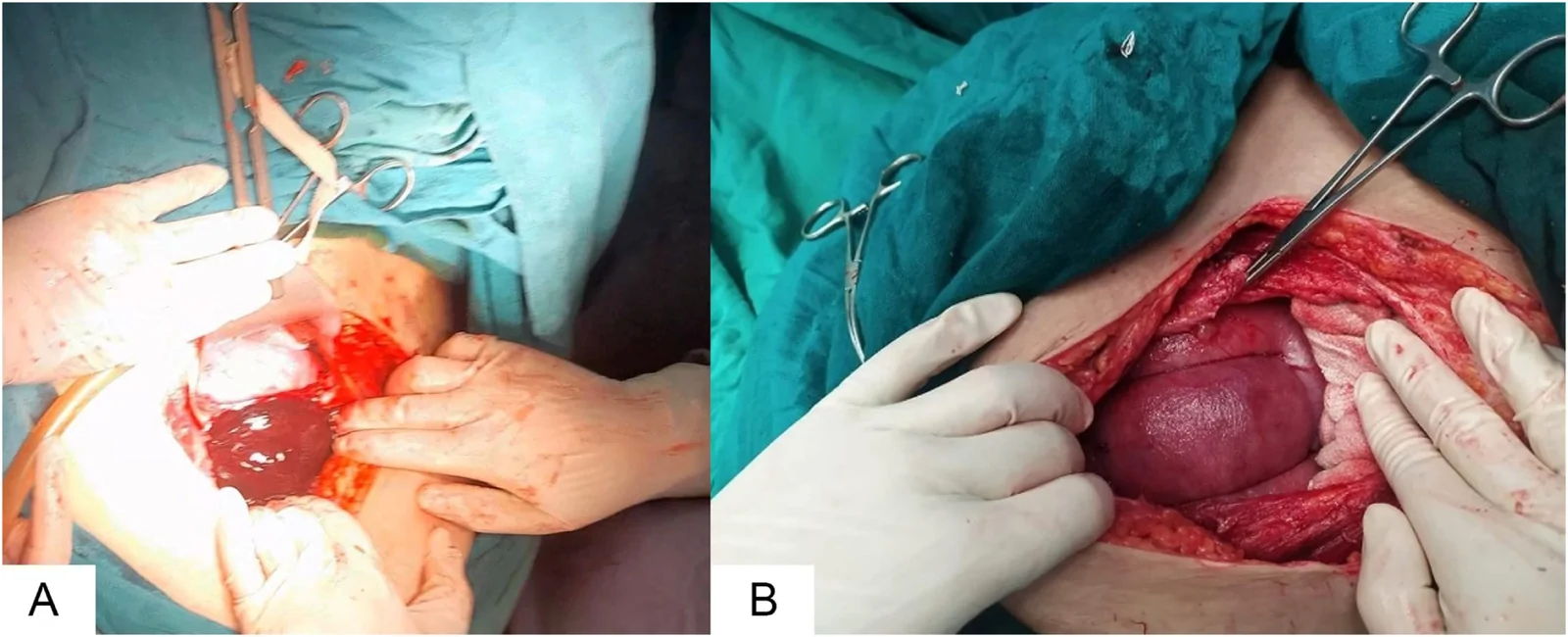
Intraoperative surgical findings and management. (A) Emergency cesarean section exposure demonstrating a dual-generation hemorrhagic event. The extensive, organized dark purple-to-black clots represent the 2-month chronic abruption, while the superimposed fresh, bright crimson-red clots confirm the acute-on-chronic decompensation leading to maternal hemorrhagic shock. (B) Direct surgical exposure showing diffuse bluish-purple myometrial ecchymosis consistent with a Couvelaire uterus. The photograph captures the placement of a uterine compression suture, which successfully controlled severe intraoperative atony and postpartum hemorrhage, thereby avoiding an emergency peripartum hysterectomy.

**Figure 3.**
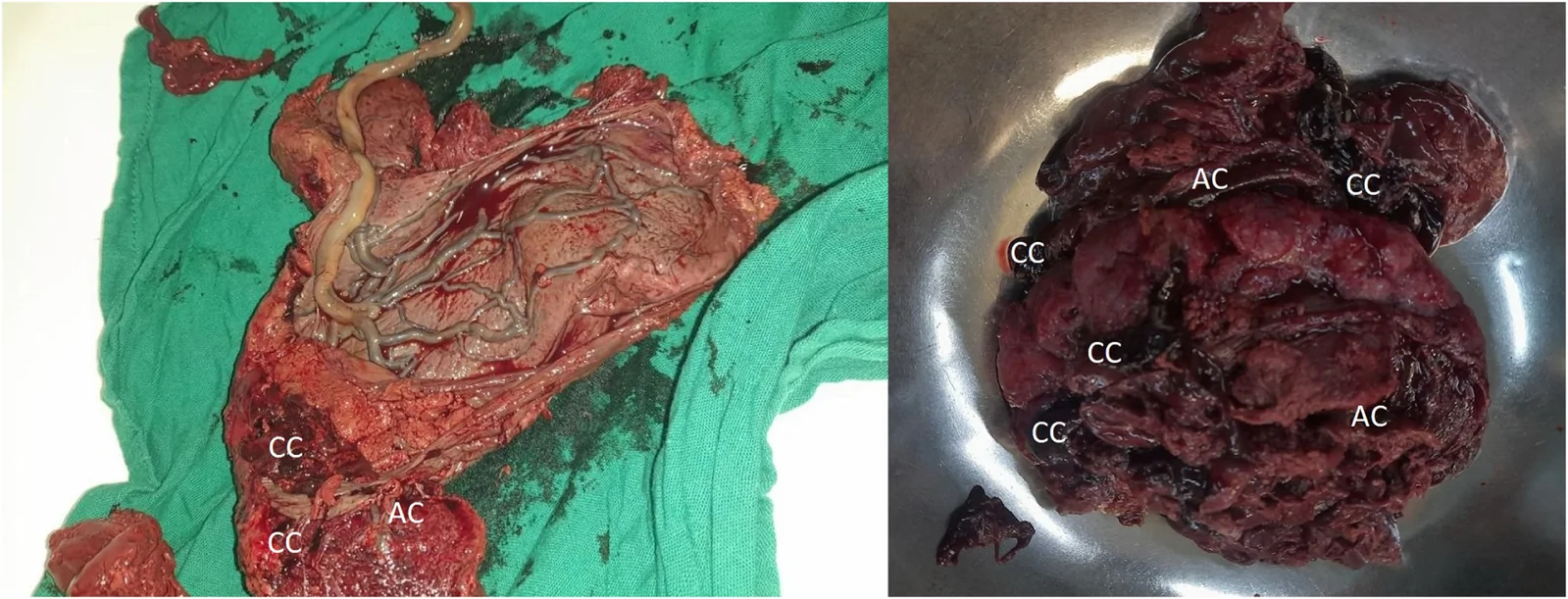
Gross pathology of the placenta in a case of chronic placental abruption. Chronic clots (CC): The label (CC) points to large, dense, dark purple-to-black consolidated clots. These represent long-standing, old, organized hemorrhage that exerted progressive mechanical pressure on the underlying placental tissue over a 2-month period. Acute clots (AC): The label (AC) highlights distinct, interspersed areas of fresh, bright, crimson-red clots that are soft and friable. These represent sudden, superimposed acute hemorrhage that led to clinical decompensation, a rapid drop in hemoglobin, and maternal hemorrhagic shock.

Intraoperatively, uterine atony with significant hemorrhage occurred. This was managed by placement of a uterine compression suture. Throughout the surgical intervention, aggressive resuscitation was maintained, and the patient received an additional 5 units of cross-matched packed red blood cells, matched to her blood group, along with 3 units of fresh frozen plasma to restore hemodynamic stability. Postoperatively, the patient was closely and continuously monitored in the ward for a total of 10 days. Her recovery was uneventful, demonstrating a well-contracted uterus and stable maternal vital signs, as supported by improved discharge laboratory parameters (Table 1). The patient was discharged in good health and continues to undergo regular, periodic follow-up evaluations in the outpatient clinic, demonstrating sustained recovery without long-term maternal complications to date.

## Discussion

Acute-on-chronic placental abruption represents a distinct and clinically challenging entity characterized by a sudden, catastrophic acute exacerbation superimposed on a pre-existing, low-grade decidual hemorrhage. In chronic cases, persistent intrauterine bleeding can progressively impair placental function, leading to amniotic fluid depletion and oligohydramnios in the absence of premature rupture of membranes (PROM). This pathology is typically recognized when a patient exhibits a prolonged history of recurrent vaginal bleeding alongside a sonographically confirmed retroplacental or subchorionic hematoma, followed by the secondary depletion of amniotic fluid. The case presented herein aptly exemplifies the classic yet extreme spectrum of an acute-on-chronic manifestation, notable for its extraordinarily prolonged chronic phase lasting 2 months, which abruptly culminated in profound maternal hemodynamic compromise and life-threatening hemorrhagic shock.

The underlying pathophysiology involves persistent, low-grade decidual bleeding that triggers localized inflammation, thrombin deposition, and progressive placental insufficiency. In our case, despite a remarkable 2-month history of chronic placental separation, serial antenatal imaging was unavailable. Emergency ultrasonography performed upon arrival at 28 weeks’ gestation clearly demonstrated oligohydramnios alongside a retroplacental hematoma, supporting an acute-on-chronic placental process.

This clinical picture underscores a highly effective feto-placental compensatory mechanism during the chronic phase. The unaffected, functioning portion of the placenta was capable of sustaining sufficient respiratory and metabolic exchange to prevent acute fetal hypoxia until the sudden acute exacerbation abruptly overwhelmed these compensatory boundaries and necessitated immediate surgical delivery.

Furthermore, the preservation of linear cranial growth was objectively demonstrated by a normal biparietal diameter (BPD) of 65.8 mm (Fig. 1c). While the remaining placental area sufficed to maintain basic fetal metabolic demands, the cumulative chronic ischemia severely compromised fetal renal perfusion. This reduction in renal blood flow led to diminished fetal urine production, which is the primary component of amniotic fluid in the second half of pregnancy, resulting in severe oligohydramnios and marked fetal crowding (Fig. 1b,d).

A hallmark of this case is the macroscopic evaluation of the placenta post-delivery, which provides a tangible pathological map of the disease’s chronological progression (Fig. 3). The maternal surface of the placenta clearly displayed two distinct, co-existing generations of hematomas: dense, dark, and highly consolidated clots alongside fresher, bright, crimson-red clots. The predominant dark, organized clots substantiate the prolonged 2-month history of decidual bleeding. Over time, these old hematomas exerted continuous mechanical pressure on the placental tissue, inducing chronic focal infarctions and areas of parenchymal compression, which further propagated placental insufficiency, supporting an acute-on-chronic placental abruption.

The optimal management of a prolonged, chronic placental abruption presents a profound clinical dilemma, requiring a careful balance between prolonging gestation to achieve fetal maturity and mitigating the inherent risk of sudden maternal decompensation. While a vigilant expectant approach is initially favored under strict clinical surveillance, the presence of recurrent significant bleeding poses an ongoing challenge. Rather than routinely advocating for early pregnancy termination, interventions must be strictly individualized. Clinicians must weigh the risks of extreme prematurity against the potential for sudden maternal-fetal compromise, reserving active delivery for cases with progressive hemodynamic instability, severe oligohydramnios, or acute fetal distress. In our patient, the administration of tranexamic acid and the repeated transfusion of 6 units of fully cross-matched packed red blood cells over a 2-month period temporarily stabilized maternal parameters. However, this substantial hematological support underscores the progressive, unstable nature of the retroplacental hematoma. Given the life-threatening transition to profound hemorrhagic shock observed at 28 weeks’ gestation, this case suggests that, in the presence of escalating transfusion requirements and severe oligohydramnios, a lower threshold for termination of pregnancy should be strongly favored to safeguard the mother’s life, rather than persisting with prolonged conservative measures.

However, the sudden clinical deterioration at 28 weeks, marked by severe uterine contractions, torrential vaginal bleeding, and rapid progression into maternal hemorrhagic shock (Hb 7.1 g/dL; Table 1), correlates pathologically with the fresh, bright red clots. This indicates a severe, acute-on-chronic abruption event. The long-standing, unstable retroplacental space likely predisposed the placental edge to a massive, acute vascular rupture. This superimposed acute detachment rapidly overwhelmed maternal hemodynamics, transforming a previously compensated, latent condition into an obstetric emergency that threatened both maternal and fetal survival. In long-standing placental separation, the myometrium is subjected to chronic exposure to retroplacental blood and degradation products. This persistent infiltration impairs myometrial contractility, creating a pathological state similar to a Couvelaire uterus (Fig. 2b), a well-documented complication in severe cases of abruptio placentae^[^4^]^. For future pregnancies, the patient was advised to undergo early antenatal surveillance, including serial ultrasonography. The necessity of a uterine compression suture underscores the high risk of postpartum hemorrhage (PPH) associated with prolonged chronic abruption, which demands immediate and aggressive surgical preparedness. Furthermore, this catastrophic presentation and the critical need for swift access to achieve hemostasis justify the selection of an emergency exploratory laparotomy during an emergency cesarean rather than a minimally invasive laparoscopic approach.

Regarding the neonatal outcome, the premature delivery at 28 weeks was unavoidable due to maternal life-threatening hemorrhagic shock. While the fetus demonstrated remarkable resilience by maintaining a normal heart rate and viability during the 2-month abruption period, the combination of extreme prematurity, severe mechanical compression from oligohydramnios, and the acute hypoxic insult during the final detachment led to severe respiratory distress syndrome (RDS) requiring immediate resuscitation and admission to an incubator. The basis for the reported irreversible neurological injury stems from this prolonged, compound hypoxic-ischemic exposure, in which chronic placental insufficiency was acutely compounded by severe intrapartum asphyxia in the setting of premature cerebral vulnerability.

## Conclusion

Acute-on-chronic placental abruption represents a highly volatile clinical entity that carries an unpredictable and exceptionally high risk of sudden maternal-fetal morbidity. This case critically demonstrates that seemingly stable maternal parameters and fetal viability achieved under prolonged conservative management may obscure a progressively unstable retroplacental space. When managing cases presenting with chronic placental separation with secondary prolonged oligohydramnios, clinicians must remain extremely vigilant, recognizing that aggressive hematologic support may stabilize systemic parameters without mitigating the underlying risk of catastrophic placental detachment. In the presence of escalating transfusion requirements and persistent severe oligohydramnios, a lower threshold for early termination of pregnancy should be strongly favored to safeguard the mother’s life, rather than persisting with prolonged conservative measures. Furthermore, post-delivery macroscopic evaluation of dual-generation placental clots serves as a pathological record to confirm the clinical chronology of an acute-on-chronic abruption and provides crucial insights into its intraoperative management.

### Patient perspective

The patient expressed immense satisfaction and gratitude for the timely surgical intervention and the comprehensive hematological support she received, which successfully saved her life during the acute bleeding crisis.

## Data Availability

Not applicable. All relevant clinical and laboratory data are included within the published manuscript.
